# A study on the influencing factors and response strategies for young teachers from Taiwan to teach in universities in China: a push-pull-mooring model perspective

**DOI:** 10.3389/fpsyg.2023.1182982

**Published:** 2023-10-03

**Authors:** Li Wang, Cong-Jin Miao, Jian-Hong Ye, Xin Huang, Liying Nong, Weiguaju Nong

**Affiliations:** ^1^School of Continuing Education, Hainan Vocational University of Science and Technology, Haikou, China; ^2^Faculty of Maritime, Hainan Vocational University of Science and Technology, Haikou, China; ^3^Faculty of Education, Beijing Normal University, Beijing, China; ^4^Provost's office and Academic Affairs (Graduate School), Beijing Normal University, Beijing, China; ^5^School of Education and Music, Hezhou University, Hezhou, China; ^6^School of Education, Guangxi University of Foreign Languages, Nanning, China

**Keywords:** young teachers from Taiwan, teach in mainland China, teaching in higher education, push, pull, mooring

## Abstract

**Introduction:**

A growing number of Taiwanese teachers are choosing to teach at universities in mainland China, but their jobs are not always stable. Therefore, this study aims to investigate the factors infuencing young teachers from Taiwan to teach in universities in China.

**Methods:**

Twenty-seven young teachers from Taiwan with master’s or doctoral degrees who were willing to apply to work at universities in China and who were already teaching in China were invited to conduct in-depth interviews to collect research data.The interview data were coded and analyzed according to the Push-Pull-Mooring (PPM) Model.

**Results and discussion:**

The results showed that the understanding of mainland Chinese universities among young Taiwanese teachers is not entirely consistent. Taiwanese teachers who previously studied in mainland China have a more comprehensive understanding of mainland Chinese universities, and some teachers have gained a superfcial understanding through academic exchanges between the two sides and information shared by friends.However,still,7% of the teachers have no understanding at all. Most young Taiwanese teachers indicate that they do not understand the talent recruitment policies of mainland Chinese universities. The push factors that infuence young teachers from Taiwan to teach at mainland universities are: Oversupply of teachers in Taiwan, poor environment for higher education in Taiwan, poor articulation of the cross-strait academic system, and four aspects of teacher retirement and re-employment in Taiwan. The pull factors are: Benefcial policies, salary, living environment, educational advantages and cultural dissemination in 5 areas. Mooring factors are divided into 3 aspects: personal factors, environmental factors and social factors.

## 1. Introduction

In recent years, as China has emerged as a global leader in the economic, scientific, and humanities fields, and with the promotion of the “double first-class university plan,” several mainland universities have been ranked among the top universities in the world. They have increased their efforts to recruit outstanding scholars to improve the quality of teaching and research at their universities, including offering olive branches to Taiwanese faculty members (Wang and Lee, 2020). On the contrary, with the growing phenomenon of childlessness in Taiwan (Tsai et al., 2021), the environment and resources for higher education development are gradually shrinking, and the number of faculty positions in universities is nearly saturated, making it difficult for even young talents with high research ability to obtain permanent teaching positions in Taiwan. Therefore, with the salary packages of university teachers in mainland China becoming closer to and even exceeding those in Taiwan, the transfer of university teachers in Taiwan to mainland China has gradually emerged, forming a population migration activity (Wu et al., 2014).

Push-pull theory is one of the most recognized and widely used theories of population migration in the world (Altbach, 1998) push-pull model states that people are subject to a combination of push and pull forces that form a complex and dynamic process of population migration. This model describes the negative factors that drive people away from their original location as push factors, such as economic downturn, employment difficulties, and poor local infrastructure (Zhang and Li, 2022). On the other hand, the positive factors that pull people to new destinations are described as pull factors, which may include factors such as better income (Biswas et al., 2019; Susanto and Akmal, 2021), employment prospects (Yeboah, 2021), better education (Juang and Schachner, 2020; Marré and Rupasingha, 2020), welfare systems (de Jong and de Valk, 2020; He et al., 2022), and good living conditions (Paloma et al., 2021). However, the original push-pull theory focused only on the negative factors at the origin (push) and the positive factors at the destination (pull) (Boyle and Keith, 2014), but this is still not enough to fully explain the influencing factors of population migration. Lee (1966) further noted that there are other barriers to intervention between the two factors of push and pull that may be overlooked by some, but may remain insurmountable for many; these are referred to as mooring factors. Therefore, mooring was integrated into the model, extending it to become the Push-Pull-Mooring (PPM) Model. This enabled the theoretical model to cover all personal, social and cultural variables that influence migration decisions, leading to a more thorough explanation of the migration phenomenon (Moon, 1995; Bansal et al., 2005). Curran and Saguy (2001) suggested that mooring factors such as migration cost, and personal and social environment can influence users’ migration behavior. The PPM model integrates perspectives from demographics, economics, education, sociology, and psychology to help researchers explore the topic from macro to micro, and from external to internal factors (Liao et al., 2019; Bhattacharyya and Mandke, 2022; Ghufran et al., 2022).

Cross-strait population migration has long been an important research topic in East Asia. Hong and Chang (2018) pointed out that for education policy to be effective in creating a push, it must be agreed upon by education policy participants. Therefore, identifying the factors that affect teaching in mainland universities from the perspective of the participants (Taiwanese mainland teachers) will enable more effective planning of policies and measures, which in turn will help Taiwanese teachers stay in the mainland for a long time and promote cross-strait integration. However, the current research on Taiwanese teachers teaching in universities in China has only been discussed by Wang (2019) through a literature analysis; so far, no empirical study has verified the background and its related factors that influence young Taiwanese teachers to teach in universities in China. In summary, this study aims to investigate three key questions from the perspective of the push-pull framework, focusing on young Taiwanese teachers who are willing to teach in mainland China. It utilizes qualitative research methods to explore the following three questions: 1. What are the intentions and profiles of young Taiwanese teachers who are willing to teach in mainland Chinese universities? 2.To what extent do young Taiwanese teachers understand the academic environment in mainland Chinese universities? 3.What factors influence the decision of young Taiwanese teachers to teach in mainland Chinese universities? The goal is to address the shortcomings of existing research in terms of research methodology and the depth of analysis.

## 2. Methods

This study used qualitative research methods to collect data through semi-structured interviews. Interviews allow interviewees to elaborate on events, processes, perceptions and opinions, and based on interviewees’ responses, the interviewer (moderator) can continue the interview in depth to obtain more new information (Muzari et al., 2022). Thus, qualitative interviews provide researchers with the opportunity to explore respondents’ experiences and issues in depth, thereby gaining insight into their experiences and perceptions of the topic. Interviews are thus considered one of the most important data collection tools in qualitative research (Coleman, 2022) Semi-structured personal in-depth interviews allow interviewers to delve into social and personal issues, which is relevant given the lack of previous research related to young Taiwanese teachers teaching in universities in China. Therefore, this study used semi-structured interviews to conduct relevant data collection and analysis (see Table 1).

**Table 1:** Information of interviewes teaching in China.

No	Gender F/M	School N/P	Area	Subject	Place of study	Marital status M/S
A1	F	N	West	Art, Law	China	M
A2	F	N	North China	Music Studies	Great Britain	S
A3	F	P	North China	Music Studies	China	M
A4	F	N	North China	Music Studies	Great Britain	S
A5	M	N	Central China	Computer Science	Taiwan	M
A6	M	N	Central China	Music Studies	Taiwan	S
A7	M	N	North China	Music Studies	United States of America	M
A8	F	N	South China	Music Studies	Taiwan	M
A9	F	N	North China	Choreography	China	S
A10	M	N	North China	Choreography	Taiwan	S
A11	M	N	North China	Education	Taiwan	S
A12	M	P	North China	Music Studies	Taiwan	M
A13	M	N	East China	Music Studies	China	S
A14	M	P	South China	Management Science	France	M

F, Female; M, Male; N, National; P, Private; M, Married; S, Single.

### 2.1. Participants

In this study, young Taiwanese teachers with master’s or doctoral degrees who are willing to apply for jobs in universities in China and who are already teaching in China were invited as interviewees. Young teachers in this study are defined as teachers with less than six years of teaching experience and prospective teachers who have earned a degree but are not yet teaching. Moreover, in addition to graduating from public and private colleges and universities in Taiwan (see Table 1), the respondents’ selection criteria also included Taiwanese teachers who had graduated from universities in mainland China or overseas (see Table 2).

**Table 2:** Information of interviewees who wish to teach in China.

No.	Gender F/M	School N/P	Area	Subject	Place of study	Marital status M/S
N1	M	P	Taiwan	Physical education	Taiwan	M
N2	M	N	Central China	Marine science	Australia	M
N3	M	P	Taiwan	Education, Physical education	Taiwan	S
N4	M	P	Taiwan	Physical education	Taiwan	S
N5	F	N	North China	Archeology	China	S
N6	F	N	North China	Pharmaceutical sciences	China	S
N7	M	P	Taiwan	Statistics, Education	Taiwan	M
N8	M	P	Taiwan	Business studies	Taiwan	S
N9	F	N	Taiwan	Education	Taiwan	M
N10	F	N	Taiwan	Education	Taiwan	S
N11	F	N	Taiwan	Business studies	Taiwan	S
N12	F	N	North China	Management science	Taiwan	M
N13	M	N	Taiwan	Management science	Taiwan	S

F, Female; M, Male; N, National; P, Private; M, Married; S, Single.

This study used the snowball sampling method, and sampling and interviews were conducted between September and November 2022. Due to epidemic factors, online interviews (Tencent video or WeChat voice) were used, and respondents were contacted and informed of the study purpose, methodology, and personal privacy security before the interviews. Informed consent was obtained to conduct the interviews. Each interview lasted about 30 min to 1 h, and all interviews were recorded with the authorization of the interviewees. Data collection was completed when no new information was provided by the respondent and information saturation was reached. The final sample was determined to be 27 teachers, 14 of whom were already teaching in Mainland China and 13 of whom were interested in teaching in Mainland China universities.

### 2.2. Interview questions

In this study, 11 interview questions were designed after literature exploration (Table 3), the first eight of which were used to explore the respondents’ exchange experience with mainland universities, their understanding of mainland talent attraction measures and the university environment. The last three questions were based on the literature on the PPM model and aimed to explore the factors that would lead young teachers in Taiwan to choose to teach in universities in China or to stay in Taiwan to find a job, or to teach overseas. The interview questions designed for this project were reviewed for expert content validity by five scholars with expertise in educational human resource management.

**Table 3:** Interview outline.

No.	Questions
1	Have you ever had any experience in academic exchange at a mainland university?
2	Are you surrounded by people you know at mainland colleges and universities? If so, have they shared any experiences with you?
3	Are you aware of the current policy on the introduction of teachers in mainland China? What aspects do you know based on your understanding?
4	In which province on the mainland would you (want to) teach in a college or university?
5	Do you think it is easy to apply for universities in mainland China?
6	What do you think of the current academic environment in mainland universities?
7	What do you think are the disadvantages or difficulties for young Taiwanese teachers to teach in mainland universities?
8	What do you think are the advantages of young Taiwanese teachers teaching in China?
9	What do you think are the circumstances or factors that would make you choose to teach in a university in China?
10	What do you think are the circumstances or factors that would keep you from choosing to teach at a university in China?
11	What do you think are the circumstances or factors that would make you hesitate to teach in universities in China?

### 2.3. Data coding

In this study, the interview data were coded into seven codes; the first code is gender; the second code is school type; the third code is teaching status; the fourth code is interviewee; the fifth code is for influencing factors and other content; the sixth code is the interview question item number; and the seventh code is the divider. The code example “F-N-A-05-PL-05” means that this code is from a female interviewee who graduated from a public university and has been teaching in China, and is the fifth interviewee, and the text code belongs to the pull factor. A description of the specific information codes is shown in Table 4.

**Table 4:** List of data code methods.

Code sequence	Codes	Description of meaning
First code	F, M	Gender code, representing female, male.
Second code	N, P	Graduation school type code, representing national colleges and universities or private colleges and universities
Third code	N, A	This is a code for the status of the appointment, representing those who are willing and those who are already in China
Fourth code	01 to 12	Code for the respondent.
Fifth code	PH, PL, MO, OH	Push, pull, mooring and other.
Sixth code	01 to 11	Interview question item number.
Seventh code	-	The first six code categories are separated by a divider.

### 2.4. Data analysis

Grounded theory is an approach that provides new insights into problem solving and has become a frequent interpretive research method used by social science researchers to uncover the underlying social processes that shape interactions, based on systematically collected and analyzed data and with a focus on theory generation (White and Cooper, 2022; Khoa et al., 2023). Grounded theory mainly includes microanalytic information such as open coding, spindle coding, and selective coding (Ma et al., 2022; Li et al., 2023) Specifically, for data analysis, the researcher prepares by (1) interpreting the interviews, including additional information obtained during the interviews, interview notes, and interview materials; (2) reading the materials in detail to gain a complete understanding of the information obtained; (3) searching for and analyzing themes, contexts, and structures; (4) carefully interpreting the information collected; (5) understanding the information collected and discovering its relevance; and (6) discussing and interpreting the information based on theoretical knowledge, research questions, guiding frameworks, and relevant literature. Therefore, the data were analyzed using grounded theory in order to shape the specific structures, variables, and dimensions of the factors that influence young teachers from Taiwan to teach in universities in China.

### 2.5. Quality criteria

In the research process, the content of the data collected by the researcher needs to be highly compatible with the real experiences, opinions and feelings expressed by the interviewees, and the analysis results need to accurately present the researcher’s personal experience of the interviewees to avoid the researcher’s overly subjective positions and opinions, and to avoid distorting or misinterpreting the feelings and opinions of the interviewees. Researchers must therefore understand the criteria to be used to test the reliability of the data. In the case of qualitative research, credibility can be tested through four criteria: trustworthiness, veracity, criticality, and completeness (Kyngäs et al., 2020). Therefore, these four indicators were used as the standard basis for the reliability test of the study.

In order to achieve the above-mentioned reliability test, the interviews were first transcribed into verbatim transcripts, coded and analyzed, and then provided to the respondents to confirm whether the textual expressions and analysis were consistent with the respondents’ true feelings and opinions. In addition, we used data triangulation to confirm the similarities and differences between the analysis results and the results of past studies by using past literature, and then we invited three scholars with expertise in educational human resource management and qualitative research to conduct a verification of the analysis results to confirm the correctness of the criticality and the completeness of the summary of the analysis results.

## 3. Results

This section may be divided by subheadings. It should provide a concise and precise description of the experimental results, their interpretation, as well as the experimental conclusions that can be drawn.

### 3.1. The path and extent of Taiwan teachers’ understanding of mainland universities

The analysis showed that the degree of past experience accumulated by young teachers in Taiwan was shown as follows: Two respondents said they had never attended cross-strait exchange meetings nor had friends to share with, and could only receive reports from the Taiwanese media, so they did not have any experience. Eighteen respondents indicated that they had a rough understanding of mainland universities because they had participated in cross-strait conference exchanges and academic visits, as well as the sharing of friends and relatives, and even short-term work experience in mainland China. In addition, Seven respondents indicated that they had experience of studying in mainland China and had a more comprehensive and relatively deep understanding of mainland universities. The main way to learn about the talent introduction policy is through online searching, sharing, and browsing public websites, but a few respondents said they were not sure through which channels or ways to learn about the policy.

Based on the descriptions of the 27 interviewees, young faculty members in Taiwan generally believe that the academic environment in the mainland is characterized by high difficulty in applying for jobs, better foundation, more resources, fierce competition, strong learning atmosphere, utilitarian results, low institutional applicability, and difficulty in audits. Young teachers in Taiwan have mixed expectations about their salary and benefits for teaching in China, and a few of them even expect too much. Two respondents expected to receive an annual salary of about 120,000 to 180,000 yuan based on the standard salary package of local teachers as well as provision of accommodation and settling-in expenses; eight respondents expected to receive an annual salary of 250,000 to 280,000 yuan; one respondent believed that teachers in Taiwan are expected to receive an annual salary of 400,000 yuan when they teach in China, and one respondent even said he expected to receive an annual salary of more than 1 million yuan.

### 3.2. Taiwan teacher’s intentions and reasons for teaching in China

According to the 27 respondents, most of them wanted to teach in first-tier cities such as Beijing, Shanghai, Guangzhou and coastal areas such as Fujian, while five respondents wanted to teach in other inland areas because they wanted to stay with their families or because of their past connections. The reasons for teachers from Taiwan to teach in China are, by and large, future expectations, referrals from others, retirement, and re-employment, change of working environment, and maintaining marriage.

### 3.3. Factors influencing Taiwan teachers to teach in China

By repeatedly reading and comparing the interview results, the coding was completed using open coding, spindle coding, and selective coding procedures according to the grounded theory research paradigm, and the findings were organized according to the PPM model to reveal the push, pull, and mooring factors that influence young teachers in Taiwan to teach in China. The coding results are shown in Table 5.

**Table 5:** Push, pull and mooring factors influencing Taiwan young teachers to teach in mainland China.

Main category	Sub-categories	Labeling
Push	Oversupply of teachers in Taiwan	Taiwan has a small population base, a small number of children, saturated teaching positions, and a lack of education industry concepts.
	Taiwan’s poor higher education environment	The need to do a lot of work outside of research, deadline pressure for promotion, discipline bias, lack of conditions and resources, disrespect, xenophobia, network interference.
	Poor articulation of cross-strait academic system	Mainland teaching qualifications, academic qualifications are not recognized by Taiwan, mainland qualifications need to be certified to return to Taiwan as teachers.
	Taiwan teachers retired and rehired	Retirement, re-employment.
Pull	Policy incentives	Benefit Taiwan policy incentives: welfare benefits, preparation, special recruitment.
	Economic benefits	Good academic prospects, excellent salary and reasonable performance requirements.
	Living Environment	Close geographical ties across the Taiwan Strait, influence of family and friends, influence of study and work experience, no need to be responsible for administrative work.
	Educational Advantages	The mainland recognizes the ability of Taiwanese teachers and respects them.
	Cultural Communication	Dissemination of musical instruments, and a trip to China.
Mooring	Personal factors	Personal limitations and high adaptation costs.
	Environmental Factors	Family environment: family living together, sustaining marriage, caring for parents, children’s education.
		Professional environment: lower than expected salary, highly unstable job, difficulty in assessing job title, cultural upbringing difference, research cultural difference, teaching cultural difference, school teacher expectation gap, future education development is limited, living material conditions are limited, living culture and habits difference, climate and food difference, administrative affairs are difficult to handle, interpersonal communication is not good.
		Living environment: unstable cross-strait relations, security threatened, life affected, delicate political atmosphere on both sides of the Taiwan Strait, speech constrained, Taiwanese documents and life management system not quite compatible, Taiwanese nationals cannot be a counselor, cannot have a “public servant card,” cannot participate in important performances, easy to have “double standard” treatment.
	Social factors	Complicated political climate, inadequate social security, insufficient recruitment platform, negative impact of the epidemic, lack of project support.

#### 3.3.1. Push factors of Taiwanese teachers to teach in China

The push factors to teach in China in this study refer to the negative factors that drive young Taiwanese teachers away from their original location (Taiwan). From the interviews, we summarized that the push for teaching in China includes four parts: “oversupply of teachers in Taiwan,” “poor environment of higher education in Taiwan,” “poor articulation of the cross-strait academic system,” and “retirement and re-employment of teachers in Taiwan.”

First, this study found that Taiwan’s population base itself is small and the phenomenon of childlessness is serious (Ho and Chen, 2021), causing a reduction in the number of university students, eventually leading to the saturation of university teaching positions. In addition, the lack of the concept of an education industry cannot solve the problem of shrinking teaching scale, eventually resulting in the oversupply of teachers in Taiwan; In other words, the number of teaching positions in Taiwanese universities is already adequate, and with a decrease in student enrollment, coupled with the inability to attract more students from outside of Taiwan to fill the gap, the demand for teaching positions has decreased. This could potentially lead to an oversupply of teaching staff. Therefore, the chances of young Taiwanese PhDs wanting to find teaching positions in Taiwan are very severe, as a result, they have to seek job opportunities outside of Taiwan which is the most important push force forcing young PhDs to leave Taiwan, consistent with the findings of Wang (2019).

*Taiwan has very few opportunities (A-11-M-N-PH-04).*

*Taiwan does not have the concept of industrialization of culture and education to improve the problem of fewer students and shrinking schools (A-14-M-P-PL-03).*

Second, research shows that the poor environment of higher education in Taiwan is also an important push factor, mainly in the form of bias against going to a private instead of public school or graduating from different institutions and entry-level colleges and universities, the lack of teaching conditions and resources, as well as the lack of respect and exclusion felt by those who do not speak the Minnan dialect, and the interference of personal connections in the evaluation and promotion of faculty positions in Taiwan, all of which constitute a push factor for teachers to leave Taiwan.

*Because you are hired today to work at a private school in Taiwan, no matter what, you are required to be a tutor, an administrator, in charge of admissions, and more, besides the traditional teaching and research services (N-3-M-P-PH-09).*

*I think there may be some obstacles for fresh graduates like us. I just graduated from some private universities (top ranked), not a (national) top ranked school; the bias against me going to private university exist. The resources and information we get is not the same (N-8-M-P-PH-09).*

The study further pointed out that the poor environment of higher education in Taiwan and the poor interface of the cross-strait academic degree system are also push forces. Cheng and Chang (2021) mentioned the same results, pointing out that teachers in Taiwan need to do a lot of administrative, teaching, counseling, and other tasks other than research, and still need to bear the pressure of deadline promotion, as well as the exhausting academic accreditation system, which all form important push forces in the recruitment process at the Taiwan end. In other words, Taiwanese teachers often hold multiple positions and face significant pressure for promotion. In comparison to the abundant and relatively relaxed job opportunities in Mainland China, the academic environment in Taiwanese universities has formed a tremendous driving force, pushing young Taiwanese teachers to seek teaching opportunities outside of Taiwan; Furthermore, educational qualifications between Mainland China and Taiwan are not completely interchangeable. It requires a complex process of certification to validate qualifications obtained in one region for recognition in the other. As a result, some Taiwanese young professionals who acquire educational qualifications in Mainland China are more inclined to pursue employment opportunities directly within Mainland China. In addition, the number of teachers worldwide choosing to retire early is increasing year by year (Muhammud et al., 2022), and this phenomenon is no exception in Taiwan, which undoubtedly gives teachers the opportunity to choose their jobs again, creating a certain degree of push to leave the country.

*Taiwan is actually not quite willing to recognize mainland qualifications, even if it is Peking University; no matter what the explicit text says, the actual operation is another matter (N-5-F-N-PH-09).*

*I came to Xiamen and Zhangzhou to teach after I retired (A-14-F-N-PH-04).*

#### 3.3.2. Pull factors of Taiwanese teachers to teach in China

The pull factors to teach in China in this study refer to the positive factors that pull young Taiwanese teachers to a new destination (the mainland). From the text of the interviews, we summarized that the pull to teach in China includes five aspects: “policy incentives,” “economic benefits,” “living environment,” “educational advantages,” and “cultural transmission.”

First, this study shows that incentives from Taiwan-friendly policies are important pull factors, including special welfare benefits offered to Taiwanese teachers individually by society or universities, and the establishment of special recruitment for Taiwanese teachers. This is consistent with Wang’s (2019) statement that after “Article 31 of Taiwan Benefit” was announced, the provinces have been following up, refining and gradually implementing measures to deepen the implementation of research results evaluation, appraisal of the staffing and title assessment initiatives, forming a pull of Taiwan-friendly policies attracting Taiwan teachers to teach in China.

*At first, I passed the exam and signed an annual salary contract system; the first three years of annual salary was 450,000, then the second appointment period was an annual salary of 280,000. The first appointment period was higher because I got the Fujian Province high-level talent introduction fee of 170,000; this is an additional subsidy from Fujian Province (A-8-F-N-PL-04).*

*My understanding at the time should be that they will set up a small venue specifically for Taiwanese doctoral or master’s students; that is, several schools were recruiting. I have the impression that Zhejiang Province and Fujian Province universities recruit more, and then you can go to submit your resume (A-1-F-N-PL-09).*

The second important pull factor found in this study is economic benefits, which includes three aspects: good academic prospects, good salary and reasonable performance requirements. The analysis of interview data revealed that most of the young teachers in Taiwan tend to choose universities in the north, Guangzhou, Shenzhen, and coastal areas where the economy is highly developed, and the rapid economic development of these cities has a strong “absorption” effect on people moving to the cities (Yang et al., 2020; Niu, 2022). Clearly, the above regions can provide teachers with higher salaries, job and life security, better platforms, more opportunities and resources, and broader development prospects; the ability to achieve both higher salaries and expect better academic prospects creates a great pull. Welfare expansion plays a key role in population migration activities (Sun et al., 2022), as people seek a better quality of life. People migrate for a better quality of life and economic value (Xiong et al., 2020; Oliinyk et al., 2021). In recent years, as China’s economy continues to improve, the recognition of mainland universities has increased, and mainland universities are a new environment with many possibilities for Taiwanese teachers, as well as a way to broaden their network by coming to teach on the mainland (Shi and Rao, 2010; Wood et al., 2023). The analysis further shows that the performance requirements are reasonable, with three types of performance standards for research, teaching, and part-time teaching based on their job content, and that they can set reasonable and acceptable performance requirements based on the actual situation. Taking all of the above into consideration, when Taiwanese young teachers choose to teach in Mainland China, they can not only receive generous salary and benefits but also ensure their livelihood; In addition, they can also gain better opportunities, access to more resources, and a promising platform, which greatly increases the possibility of achieving substantial academic accomplishments in the future; Furthermore, the performance requirements are relatively reasonable, creating a strong pulling force for Taiwanese young teachers to teach in Mainland China.

*I hope to have a larger platform that can make my results better and can play to my strengths and then try to make up for some of my weaknesses, which is my priority factor (A-11-M-N-PL-04).*

*In fact, there are still more opportunities in mainland China (A-1-F-N-PH-04).*

*Considering the space for development and the overall academic resources, the school where I am teaching now (in mainland China) is a better place for me use my skills (A-7-M-N-PL-04).*

This study shows that living environment is the third important pull factor, mainly referring to the close geographic relationship between the two sides of the Taiwan Strait, with many commonalities in terms of transportation distance, lifestyle, language, climate, etc. It encourages Taiwanese teachers to quickly adapt to the living environment in Mainland China; The successful development of relatives and friends in Mainland China attracts Taiwanese youth to go there. At the same time, they provide significant support to Taiwanese youth who go to Mainland China, both materially and emotionally. This creates a positive influence from family and friends, encouraging them to pursue opportunities in Mainland China. The attraction and support of family and friends to develop in the mainland has generated the influence of family and friends, and the experience of studying and working in the mainland has made people adapt to the mainland life and have a positive attitude. Not being responsible for administrative work gives Taiwanese teachers more personal free time, allowing them to better organize their lives and work, as well as reducing communication with school supervisors, avoiding conflicts of interest and friction, and having a simple work environment.

*And they understand Chinese, the language is similar, Taiwan will be more similar, so they may go (N-1-M-P-PL-08).*

*Because my husband is from Shanxi, so I hope to return to Shanxi to teach (A-1-F-N-PL-04).*

*I’ve been on the mainland for so long that I’ve gotten used to it, so I ended up staying on the mainland to teach in universities (A-13-M-N-PL-09).*

Furthermore, Mainland Chinese universities recognize the strengths of Taiwanese teachers. The traditional value of respecting and honoring teachers in Mainland China has formed an advantage in teaching, which attracts Taiwanese young teachers to go and teach in Mainland Chinese universities. The mainland’s tradition of respecting teachers form a teaching advantage that pulls teachers to teach in mainland universities. This study found that Taiwan’s master’s degree takes longer to develop, requires academic exchange and overseas exchange experience, and undergoes rigorous academic training, resulting in solid research skills that become one of the strengths of Taiwan’s faculty. In addition, a quarter of Taiwan’s colleges and universities are ranked among the world’s top universities, and graduating from them makes teachers highly visible, which is the second advantage of teachers in Taiwan. Moreover, the analysis shows that Taiwanese teachers are enthusiastic in teaching, flexible in teaching style, broad in international perspective, friendly and harmonious with students and teachers as well as friends, and young teachers are tolerant and malleable, which form the strengths of Taiwanese teachers (Campbell, 2003; Li, 2022). Moreover, mainland schools cooperate with the needs of teachers, teachers respect each other, and students respect teachers, forming a good tradition of respect for teachers (Lan and Moscardino, 2019). The recognition of the inherent strengths of Taiwanese teachers by Mainland Chinese universities, as well as the positive traditions of higher education institutions in Mainland China, together constitute important driving factors for Taiwanese teachers to choose to teach in Mainland China.

*I think there are several advantages for young teachers from Taiwan to teach in mainland China, which are relatively few. For example, doing empirical research may be a little stronger.. we write journal articles of empirical research. There are 16 schools in Taiwan ranked among the world’s top universities (A-11-M-N-PL-08).*

*We can be teachers and students in class and friends at the end of class, and that in itself is needed (A-13-M-N-PL-08).*

*Mainland students are very respectful of their teachers and have a certain ethical standard (A-2-F-N-PL-07).*

Finally, cultural transmission is the fifth pull factor in this study (Asselin and Drainville, 2020). Mainland China is a vast and culturally diverse country. In particular, teachers of the arts can come to the mainland to teach both to collect and to spread musical instruments or art culture. Undoubtedly, the opportunity to engage in cultural exchange and dissemination is an attractive aspect that can draw Taiwanese young teachers to go to Mainland China.

*For me it was most direct to come to the mainland to collect stories, so I was willing to come over, so in 2012 I arrived in Shanghai (A-6-M-N-PH-09).*

*From the beginning, I chose to teach in mainland universities because I wanted to do art education to promote musical instruments and culture (A-8-F-N-PH-09).*

#### 3.3.3. Mooring factors of Taiwanese teachers to teach in China

The mooring force for teaching in China in this study refers to the barrier factors that prevent young Taiwanese teachers from leaving their original place of residence (Taiwan) and going to their new destination (mainland China). From the text of the interviews, we summarize that the mooring force of teaching in China is divided into three aspects: personal factors, environmental factors, and social factors.

First, personal factors are the barrier factors that affect Taiwanese teachers to teach in China, including both personal limitations and higher adaptation costs. The analysis revealed that the psychological or emotional discomfort that young teachers in Taiwan may experience as a result of leaving their former familiar environment and identity not only incurs relationship transition costs (Christino et al., 2020; Demir et al., 2021), but also generally requires the re-establishment of new network resources and overcoming psychological adjustment barriers, which adds to the adaptation costs. In addition, young teachers in Taiwan focus on immediate benefits and are prone to hesitate, retreat, or even lose out on small things once the benefits do not meet their expectations or are compromised. They like to seek the stratosphere and are willing to complain about the bad adaptation process; they are also prone to inexplicable feelings of superiority and overconfidence. Therefore, the above-mentioned personal constraints and high adaptation costs are all personal factors that prevent young teachers from teaching in Taiwan.

*I think that young teachers in Taiwan focus too much on their own interests and may actually lose a little for the sake of a little (A-2-F-N-MO-08).*

*I think Taiwanese people like to complain about everything, but they are actually unable to make real changes (A-2-F-N-MO-07).*

*No experience (very few sources of information), they are sometimes worried about going over to the mainland without knowing anything (N-13-M-N-MO-07).*

Second, environmental factors are obstacles that prevent Taiwanese teachers from teaching in China, including family environment, professional environment, and living environment. Although Fan and Li (2019) have confirmed that a “core family” approach is prevalent in China’s population migration, with families moving with them to their destinations in order to maintain their marriages, discipline their children, and even care for their elderly parents, married teachers, who are unable to migrate their entire families, are severely hindered from teaching in China (Bunn et al., 2022).

*If I get married, I may hesitate to follow my significant other to live in the city where he works if he does not work in mainland China (A-9-F-N-MO-11).*

*I am new to teaching; if there is a sudden change in my family, such as an unhealthy elder or something, this is the only reason why I would hesitate to come to the mainland China to teach (A-2-F-N-MO-11).*

*I think the only reason why I hesitate to teach in China is because of my children’s education (A-3-F-P-MO-11).*

Despite the incentive of “Taiwan-friendly” policies, some of these policies may not be effectively implemented due to the intensified political climate across the Taiwan Strait or other special reasons. Moreover, the actual salary may be lower than expected. In addition, most universities set the situation of age limit for application for employment and use the employment system (no state establishment) to hire young Taiwanese teachers, usually with short-term agreements of 2–5 years. The increased job instability contributes to higher staff turnover (separation rate) (Zhou and Zhong, 2022). Moreover, unlike Taiwan, there is a quota control in the actual evaluation of university teachers’ titles in mainland China due to the overall national system; that is, many qualified teachers compete for a limited number of places, so the “in-roll” is very serious (Mulvey and Wright, 2022), and obviously the title evaluation is difficult. Next, there are differences in cultural upbringing and research culture between the two sides of the Taiwan Strait, with mainland education emphasizing subject systematization and in-depth training, while Taiwan focuses more on international training. It is clear that culture will also influence teacher integration (Sun et al., 2022). As the phenomenon of fewer children on the mainland is becoming increasingly serious (Marois et al., 2021), some Taiwanese teachers are also concerned that future teaching positions in mainland universities will also be saturated, and once the staff is cut, there is a greater possibility that Taiwanese teachers will be laid off, so their career development is limited. As mentioned above, the career environment of low salary expectations, short-term employment instability, difficulties in title evaluation, cultural differences and future career restrictions are hindering young Taiwanese teachers from teaching in the mainland.

*In fact, the entanglement treatment is not enough for the situation, … There is a difference from the original imaginary life (N-7-M-P-MO-10).*

*If the subject gives you 80,000 yuan, you can use the 80,000 yuan any way you want. However, how can you publish a paper if you cannot audit the publication charges? It would be financially stressful for me to have the money and not be able to use it (A-11-M-N-MO-06).*

Analysis shows that living environment factors are also obstacles for young teachers in Taiwan to teach in China, such as living material conditions, differences in living culture and habits, climatic and dietary differences, difficulties in handling administrative matters, and poor interpersonal communication.

*I think the other difficulties are that it is not very friendly administratively. I think the administrative process is very complicated for me, and the ID of Taiwanese or residence permit is not the same as the ID card of mainland China and may not be coherent (not connected) with some application systems (A-11-M-N-MO-07).*

*I think the people in mainland colleges and universities only care for themselves (very egotistical). There is no warmth between people (A-8-F-N-MO-06).*

Finally, social factors also hinder young teachers from Taiwan to teach in China, including five aspects: a complicated political climate, insufficient social security, inadequate recruitment platform, negative impact of the epidemic, and lack of program support. Unstable cross-strait relations and a delicate political climate make Taiwan teachers feel that their safety may be threatened, their livelihoods affected to some extent, and their speech restrained. In addition, it also makes Taiwan close the channels and online platforms for recruiting talent from the mainland, resulting in a lack of channels for Taiwan teachers to learn about university recruiting information. However, the social security provided by society to young Taiwanese faculty members is inadequate, such as the failure of some Taiwanese scholars to move into on-campus specialist buildings, swing houses or family homes, the lack of transitional jobs for accompanying family members, and inadequate medical coverage. In addition, the mainland China’s tax rate is too heavy, which seriously hinders young Taiwanese teachers from seeking jobs and settling down in the mainland. During the special period, the COVID-19 epidemic made it difficult to enter and leave the country and hindered the return to Taiwan to visit relatives. Even so, there are many Taiwan teachers appealing to national and provincial projects lacking high-level Taiwanese titles and special topics, hoping that the state will provide support to assist them in adapting to teaching and research in mainland universities as soon as possible. The various social aspects described above will play a very significant role in hindering young teachers from Taiwan from teaching in China.

*I think political instability is a factor that makes me doubt whether to teach in China (N-13-M-N-MO-10).*

*Because without this pipeline, it is not possible to learn a lot of news, which is also more difficult (A-1-F-N-MO-07).*

## 4. Discussion

### 4.1. Findings

Based on the analysis of the results, it is evident that the understanding of mainland Chinese universities among young Taiwanese teachers is not consistent. Taiwanese teachers who previously studied in mainland China have a more comprehensive understanding of mainland Chinese universities. Some teachers have gained a superficial understanding through academic exchanges between the two sides and information shared by friends. However, there is still 7 % of the participants who have no understanding of mainland Chinese universities at all. Most young Taiwanese teachers indicate that they are not familiar with the talent recruitment policies of mainland Chinese universities. This is primarily due to limited channels for obtaining information, internet censorship, and information asymmetry. Clearly, attracting Taiwanese students to study in mainland China, enhancing academic exchanges between the two sides, and promoting cross-strait social interactions are important avenues for Taiwanese youth to gain knowledge of, understand, identify with, and integrate into mainland Chinese universities and culture (Chou and Ching, 2020; Zhang et al., 2020; Schubert et al., 2021). Furthermore, establishing effective channels for promoting awareness of mainland Chinese university talent recruitment policies is crucial for enhancing the in-depth understanding of pro-Taiwan policies among Taiwanese youth. Sincere and friendly treatment of Taiwanese teachers who come to mainland China and building a positive reputation are also of utmost importance.

This research demonstrates that whether young Taiwanese teachers choose to teach in mainland China is influenced by the interplay of push factors, pull factors, and anchoring forces (Huang et al., 2022). The push factors primarily stem from certain negative conditions in Taiwan, including an oversupply of teaching staff, an unfavorable higher education environment, difficulties in aligning academic qualifications between the two sides, and retired Taiwanese teachers seeking employment opportunities in mainland China. The pull factors mainly consist of positive incentives offered by mainland Chinese universities to Taiwanese teachers. These factors include pro-Taiwan policies as incentives, promising career prospects, a favorable living environment, and educational advantages, among others. Despite the push and pull factors mentioned above encouraging young Taiwanese teachers to choose teaching positions in mainland China, they still take into account certain hindrances stemming from personal, environmental, and social anchoring forces (Kim, 2021). For instance, these hindrances include personal goal constraints, higher adaptation costs, family-related limitations, unsuitable career environments, a volatile living environment, complex political atmosphere, inadequate social security, insufficient recruitment platforms, negative impacts from the pandemic, and insufficient project support. Further optimizing the pull factors and minimizing or eliminating the obstacles caused by anchoring forces is a future direction for efforts to encourage young Taiwanese teachers to teach in mainland China.

### 4.2. Research contribution

This study has the following theoretical value: through interviews and grounded theoretical analysis research methods, it first clarifies the knowledge of young Taiwanese teachers about mainland universities and talent policies, as well as the degree of willingness to teach in mainland China, enriching the study of career development of Taiwanese teachers. Secondly, we constructed a push-pull mooring force model of the factors influencing young teachers from Taiwan to serve in China, and analyzed the content of three different aspects of push, pull, and mooring forces, which effectively promoted the study of the push-pull-mooring force theory in the direction of population migration and career development (Huang et al., 2022).

### 4.3. Future research

This study used a qualitative research method to explore the influencing factors of the willingness of young teachers from Taiwan to teach in China, which is of reference value for promoting the professional development of teachers from Taiwan and promoting cross-strait teacher research. However, as the research topic was limited to young teachers from Taiwan, the interviewees of this study were young teachers from Taiwan who wish to teach or who are teaching in China. In the future, the research population can be broadened to include teachers of different age groups, different countries, a particular region, and a particular profession or group. Second, as a qualitative research method was used in this study, further quantitative research methods may be adopted in the future to validate the results of this study. Third, in the future, we may also try to adopt a case or narrative study approach (Voznyak et al., 2021), focusing on a few representative Taiwanese teachers to conduct in-depth interviews and long-term observations to explore the factors that have influenced the push and pull system of Taiwanese teachers’ career development in China or on the mainland at different times and how these factors have changed.

## 5. Conclusions and recommendations

### 5.1. Conclusion

This study started from the perceived factors of pushing, pulling and mooring forces of young teachers in Taiwan to teach in colleges and universities in China, and explored the influencing factors. The aim was to understand in detail the current factors affecting the decisions of young teachers in Taiwan to teach in colleges and universities in China. Through the interview study of 27 young teachers in Taiwan who intend to teach in Mainland China or who are currently teaching in China, the results showed that about 7 % of the young teachers in Taiwan knew nothing about the universities in Mainland China, Sixty-six percent of the interviewees had a superficial understanding through cross-strait academic exchanges and friends’ sharing, and less than 25 % of the interviewees had a more comprehensive understanding because they had studied in Mainland China. Taiwan young teachers’ understanding of the policy of introducing talents to mainland universities is what most of the respondents said they did not understand, while one third said they generally understood. By analyzing, summarizing and digging deeper into the interview data, a push-pull-mooring model was established for young teachers from Taiwan to teach in China. The results showed that the push force consisted of an oversupply of teachers in Taiwan, poor environment of higher education in Taiwan, poor articulation of the cross-strait academic system, and retirement and re-employment of teachers in Taiwan. The pulling force consists of policy incentives, economic treatment, living environment, educational advantages, and cultural transmission; while the mooring force consists of personal factors, environmental factors, and social factors. The combined dynamics of the above factors influence the demographic migration of young Taiwanese teachers to teach in China.

### 5.2. Recommendations

Based on the analysis and discussion of the results of this study, in order to improve the willingness of young teachers in Taiwan to teach in China and to address the factors that are barriers to teacher integration, suggestions are made for this purpose from three aspects: individual young teachers in Taiwan, universities, and the state.

#### 5.2.1. It is recommended that Taiwanese young teachers reduce the cost of adaptation and break personal limitations in order to minimize the impact of personal restraining factors

It is recommended that young teachers in Taiwan strengthen their understanding of the mainland through cross-strait meetings and exchanges, sharing with family and friends, experiencing life, and going to the mainland to upgrade their education. Furthermore, Taiwanese teachers can reduce barriers in the adaptation process and ultimately lower the cost of adaptation by strengthening their teaching and research abilities, establishing good teacher-student relationships, and continuously enhancing their own strengths. By maintaining a long-term perspective, adopting a humble and cautious attitude, and approaching the challenges and opportunities presented by the new environment of Mainland Chinese universities with a positive mindset, Taiwanese teachers can ultimately break through personal limitations.

#### 5.2.2. It is recommended that universities improve the professional environment to reduce the impact of environmental restraining factors

The results of the study showed that the duration of doctoral formation in Taiwan is longer, and many young Taiwanese doctors who aspire to teach in mainland universities lose the opportunity to apply for jobs due to age restrictions. Therefore, the age limit is narrowed for doctoral talents with academic achievements or exceptional performance. In addition, the professional environment is an important tie-in force. In order to help first-time Taiwanese teachers adapt smoothly, it is recommended that a coaching period, an improvement period, and a parallel replacement mechanism for performance be added to the performance appraisal.

#### 5.2.3. It is recommended to enhance social security and project support to alleviate the impact of social restraining factors

Research shows that most Taiwanese teachers teach at universities in China on an annual contract basis, and although they have higher salaries and benefits than those of teachers on staff, they have less job security. Renewals may not be available due to one time performance, changes in university policy or talent attraction policies, etc. Therefore, it is suggested that the number of career teachers in Taiwan can be upgraded so that teachers who meet the performance requirements can enjoy the original salary package while having the opportunity to enjoy career status.

Research shows that most of the Taiwanese faculty members are highly expected by the appointing schools to make use of their academic expertise to publish SSCI/SCI and CSSCI journal articles and to initiate national and provincial level funding projects. However, due to the writing style and academic training of Taiwanese faculty, there is a certain degree of difficulty in applying for projects and submitting articles to C journals, and so most articles are published in international journals. Moreover, in order to conduct research, scholars still need to have project support in order to have good academic output. Therefore, it is suggested that a “special support project for Taiwanese” can be set up with reference to the Social Science Foundation of Fujian Province. In national and provincial projects such as the National Science Foundation, the National Social Science Foundation, the Ministry of Education’s Humanities and Social Science Research or the National Education Science Planning, special projects for Taiwan talents or talents from Hong Kong, Macao and Taiwan can be added to give young teachers from Taiwan more opportunities to develop their academic expertise and promote their titles.

## Data availability statement

The raw data supporting the conclusions of this article will be made available by the authors, without undue reservation.

## Ethics statement

Ethical review and approval was not required for the study on human participants in accordance with the local legislation and institutional requirements. Oral informed consent to participate in this study was provided by the patient/participants.

## Author contributions

LW, and J-HY: concept and design and drafting of the manuscript. LW, J-HY and C-JM: acquisition of data, Grounded theory coding and analysis. LW, J-HY, XH, LN and WN: critical revision of the manuscript. All authors contributed to the article and approved the submitted version.

## References

[ref1] Altbach P. G. (1998). Comparative higher education: Knowledge, the university, and development. Greenwood Publishing Group. Westport

[ref2] Asselin H. Drainville R. (2020). Are indigenous youth in a tug-of-war between community and city? Reflections from a visioning workshop in the lac Simon Anishnaabeg community (Quebec, Canada). World development. Perspectives 17:e100168:100168. doi: 10.1016/j.wdp.2019.100168

[ref3] Bansal H. S. Taylor S. F. St. James Y. (2005). “Migrating” to new service providers: toward a unifying framework of consumers’ switching behaviors. J. Acad. Mark. Sci. 33, 96–115. doi: 10.1177/0092070304267928

[ref4] Bhattacharyya S. S. Mandke P. V. (2022). Study of awareness, adoption and experience of telemedicine technology services; perspectives during coronavirus (COVID-19) pandemic crisis and associated economic lockdown in India. J. Sci. Technol. Policy Manag 13, 788–811. doi: 10.1108/JSTPM-10-2020-0146

[ref5] Biswas R. K. Kabir E. Khan H. T. (2019). Causes of urban migration in Bangladesh: evidence from the urban health survey. Popul. Res. Policy Rev. 38, 593–614. doi: 10.1007/s11113-019-09532-3, PMID: 20832832

[ref6] Boyle P. Keith H. (2014). Exploring contemporary migration. Routledge. Oxfordshire

[ref7] Bunn M. Zolman N. Smith C. P. Khanna D. Hanneke R. Betancourt T. S.. (2022). Family-based mental health interventions for refugees across the migration continuum: a systematic review. SSM-Mental Health, Article:e100153. doi: 10.1016/j.ssmmh.2022.100153, PMID: 37529116 PMC10392776

[ref8] Campbell E. (2003). The ethical teacher. Open University Press. United Kingdom

[ref9] Cheng Y. -J. Chang T.-W. (2021). Between pushing and pulling? An analysis of Taiwan's brain drain from the “push-pull theory” and how to respond to it: the example of doctoral students in the United States. J. Educ. Sci. Res. 66, 1–33. doi: 10.6209/JORIES.202106_66(2).0001

[ref10] Chou C. P. Ching G. S. (2020). Evolving international academic exchanges: the shifting cross-strait university practices between Taiwan and China. Intern. J. Res. 9, 1–9. doi: 10.5861/ijrse.2020.5014

[ref11] Christino J. Silva T. Moura L. R. Fonseca L. H. (2020). Antecedents and consequents of brand love in the smartphone market: an extended study of the impact of switching cost. J. Promot. Manag. 26, 301–321. doi: 10.1080/10496491.2019.1699630

[ref12] Coleman P. (2022). Validity and reliability within qualitative research for the caring sciences. Int. J. Caring Sci. 14, 2041–2045.

[ref13] Curran S. R. Saguy A. C. (2001). Migration and cultural change: a role for gender and social networks? J. Int. Women's Stud. 2, 54–77.

[ref14] de Jong P. W. de Valk H. A. (2020). Intra-European migration decisions and welfare systems: the missing life course link. J. Ethn. Migr. Stud. 46, 1773–1791. doi: 10.1080/1369183X.2019.1611421

[ref15] Demir A. Budur T. Heshmati A. (2021). Antecedents of trust, corporate image, and switching costs: a case in telecommunication services in the Kurdistan region of Iraq. Int. J. Mob. Commun. 19, 53–74. doi: 10.1504/IJMC.2021.111892

[ref16] Fan C. C. Li T. (2019). Familization of rural–urban migration in China: evidence from the 2011 and 2015 national floating population surveys. Area Dev. Policy 4, 134–156. doi: 10.1080/23792949.2018.1514981

[ref17] Ghufran M. Ali S. Ariyesti F. R. Nawaz M. A. Aldieri L. Xiaobao P. (2022). Impact of COVID-19 to customers switching intention in the food segments: the push, pull and mooring effects in consumer migration towards organic food. Food Qual. Prefer. 99:e104561. doi: 10.1016/j.foodqual.2022.104561

[ref18] He A. J. Zhang C. Qian J. (2022). COVID-19 and social inequality in China:the local–migrant divide and the limits of social protections in a pandemic. Polic. Soc. 41, 275–290. doi: 10.1093/polsoc/puac003

[ref19] Ho S. S-H. Chen R.J-C. (2021). Universities in the knowledge society. Springer. Berlin

[ref20] Hong J.-C. Chang R.-E. (2018). A study on teachers' acceptance attitudes toward functionally integrated teaching and learning policies in a university of science and technology-using the “industrial function benchmarking system” as a mediating environment. J. Educ. Sci. Res. 63, 251–284.

[ref21] Huang L. Huang Y. Huang R. Xie G. Cai W. (2022). Factors influencing returning migrants’ entrepreneurship intentions for rural E-commerce: an empirical investigation in China. Sustainability 14:e3682. doi: 10.3390/su14063682

[ref22] Juang L. P. Schachner M. K. (2020). Cultural diversity, migration and education. Int. J. Psychol. 55, 695–701. doi: 10.1002/ijop.12702, PMID: 32672392

[ref23] Khoa B. T. Hung B. P. Hejsalem-Brahmi M. (2023). Qualitative research in social sciences: data collection, data analysis and report writing. Intern. J. Public Sector Performance Manag. 12, 187–209. doi: 10.1504/IJPSPM.2023.132247

[ref24] Kim K. (2021). Conceptualization and examination of the push-pull-mooring framework in predicting fitness consumer switching behavior. J. Global Sport Management 12, 1–23. doi: 10.1080/24704067.2021.2013128

[ref25] Kyngäs H. Kääriäinen M. Elo S. (2020). The application of content analysis in nursing science research. Springer. Berlin

[ref26] Lan X. Moscardino U. (2019). Direct and interactive effects of perceived teacher-student relationship and grit on student wellbeing among stay-behind early adolescents in urban China. Learn. Individ. Differ. 69, 129–137. doi: 10.1016/j.lindif.2018.12.003

[ref27] Lee E. S. (1966). A theory of migration. Demography 3, 47–57. doi: 10.2307/2060063, PMID: 37672878

[ref28] Li J. B. (2022). Teacher–student relationships and academic adaptation in college freshmen: disentangling the between-person and within-person effects. J. Adolesc. 94, 538–553. doi: 10.1002/jad.12045, PMID: 35383945

[ref29] Li X. Cui W. Chee W. M. (2023). Investigating tourism experiences and attention allocation of outbound tourists through the lens of the two-factor theory: a grounded theory analysis of Chinese tourists' travelogues in Malaysia. Heliyon 9. doi: 10.1016/j.heliyon.2023.e17896, PMID: 37483812 PMC10359871

[ref30] Liao Y. W. Huang Y. M. Huang S. H. Chen H. C. Wei C. W. (2019). Exploring the switching intention of learners on social network-based learning platforms: a perspective of the push–pull–mooring model. Eurasia journal of mathematics, science and technology. Education 15:e1747. doi: 10.29333/ejmste/108483

[ref31] Ma L. Zhang X. Wang G. (2022). The impact of enterprise social media use on employee performance: a grounded theory approach. J. Enterp. Inf. Manag. 35, 481–503. doi: 10.1108/JEIM-08-2020-0331

[ref32] Marois G. Gietel-Basten S. Lutz W. (2021). China's low fertility may not hinder future prosperity. Proc. Natl. Acad. Sci. 118:e2108900118. doi: 10.1073/pnas.2108900118, PMID: 34580226 PMC8501780

[ref33] Marré A. W. Rupasingha A. (2020). School quality and rural in-migration: can better rural schools attract new residents? J. Reg. Sci. 60, 156–173. doi: 10.1111/jors.12437

[ref34] Moon B. (1995). Paradigms in migration research: Exploring'moorings' as a schema. Prog. Hum. Geogr. 19, 504–524. doi: 10.1177/030913259501900404, PMID: 12347395

[ref35] Muhammud A. Abd Aziz N. Bakar R. A. Harun S. (2022). Factors influencing the intention for early retirement: a case study among teachers at secondary cluster schools in Dungun Terengganu. Responsible Educ, Learn, and Teach Emerging Eco 4. doi: 10.26710/relate.v4i2.2483

[ref36] Mulvey B. Wright E. (2022). Global and local possible selves: differentiated strategies for positional competition among Chinese university students. Br. Educ. Res. J. 48, 841–858. doi: 10.1002/berj.3797

[ref37] Muzari T. Shava G. N. Shonhiwa S. (2022). Qualitative research paradigm, a key research design for educational researchers, processes and procedures: a theoretical overview. Indiana J, Humanities and Soc. Sci. 3, 14–20. (1)_14-20_61f38990115064.95135470.pdf https://indianapublications.com/articles/IJHSS_3

[ref38] Niu F. (2022). A push-pull model for inter-city migration simulation. Cities 131:e104005. doi: 10.1016/j.cities.2022.104005

[ref39] Oliinyk O. Bilan Y. Mishchuk H. Akimov O. Vasa L. (2021). The impact of migration of highly skilled workers on the country's competitiveness and economic growth. Montenegrin J. Eco. 17, 7–19. doi: 10.14254/1800-5845/2021.17-3.1

[ref40] Paloma V. Escobar-Ballesta M. Galván-Vega B. Díaz-Bautista J. D. Benítez I. (2021). Determinants of life satisfaction of economic migrants coming from developing countries to countries with very high human development: a systematic review. Appl. Res. Qual. Life 16, 435–455. doi: 10.1007/s11482-020-09832-3

[ref41] Schubert G. Rigger S. Zani B. Lin S. S. Chen C. J. J. (2021). Delimiting ‘Cross-Strait studies’: Kua’an vs. Liang’an. Intern. J. Taiwan studies 4, 163–191. doi: 10.1163/24688800-20201193

[ref9001] Shi Y. Rao Y. (2010). China’s research culture. Science. 329, 1128. doi: 10.1126/science.1196916 20813923

[ref42] Sun X. Chen J. Xie S. (2022). Becoming urban citizens: Athree-phase perspective on the social integration of rural–urban migrants in China. Int. J. Environ. Res. Public Health 19:e 5946. doi: 10.3390/ijerph19105946 PMC9141490 35627482

[ref43] Susanto H. Akmal H. (2021). Migration and adaptation of the loksado dayak tribe (historical study of dayak loksado community in pelantingan village). In 2nd international conference on social sciences education (ICSSE 2020) (pp. 5–10). Atlantis Press. doi: 10.2991/assehr.k.210222.002

[ref44] Tsai M. C. Peng S. C. Kuo W. B. (2021). Singlehood and childlessness: an age-period-cohort analysis of changing attitudes toward family in Taiwan (2005-2020). J. Fam. Stud. 29, 1–22. doi: 10.1080/13229400.2021.2004912

[ref45] Voznyak H. Mulska O. Bil M. (2021). Migration aspirations of territory population: a case study of Ukraine. Probl. Perspect. Manag. 19, 217–231. doi: 10.21511/ppm.19(2).2021.18

[ref46] Wang M. Y. (2019). A study on the trend of Taiwanese teachers teaching in mainland China and their integration. Taiwan Studies 5, 69–78. doi: 10.13818/j.cnki.twyj.2019.05.008

[ref47] Wang C. C. Lee L. C. (2020). Analysis of factors influencing the willingness of Taiwanese students to work on mainland China: an example of “31 preferential policies for Taiwan”. Pac. Focus. 35, 76–108. doi: 10.1111/pafo.12156

[ref48] White R. E. Cooper K. (2022). Qualitative research in the post-modern era: Critical approaches and selected methodologies. Springer. Berlin

[ref49] Wood A. Kleinbaum A. M. Wheatley T. (2023). Cultural diversity broadens social networks. J. Pers. Soc. Psychol. 124, 109–122. doi: 10.1037/pspi0000395, PMID: 35266781

[ref50] Wu C. Z. Shi N. Z. Chu J. Y. (2014). A preliminary study on the reasonableness of university professors’ salaries in Taiwan: a comparative study of university, middle and elementary school teachers. Nat. Taiwan Univ. Manag Series 24, 1–52.

[ref51] Xiong Y. Zhang Y. Lee T. J. (2020). The rural creative class: an analysis of in-migration tourism entrepreneurship. Int. J. Tour. Res. 22, 42–53. doi: 10.1002/jtr.2317

[ref52] Yang Z. Gao W. Zhao X. Hao C. Xie X. (2020). Spatiotemporal patterns of population mobility and its determinants in Chinese cities based on travel big data. Sustainability 12:e4012. doi: 10.3390/su12104012

[ref53] Yeboah T. (2021). Future aspirations of rural-urban young migrants in Accra, Ghana. Children's Geographies 19, 45–58. doi: 10.1080/14733285.2020.1737643

[ref54] Zhang K. S. Chen C. M. Hou X. X. (2020). “The discussion of communication transformation and development in cross strait higher education and technical and vocational education” in Education and awareness of sustainability: Proceedings of the 3rd Eurasian conference on educational innovation. Eds. Tijus C, Teen-Hang Meen TH and Chang CY. (Tamil Nadu: World Scientific), 829–833.

[ref55] Zhang Y. Li Q. Y. (2022). Research on the factors influencing the information cocooning willingness of internet users based on PPM theory. Modern Intell. 4, 52–61. doi: 10.3969/j.issn.1008-0821.2022.04.005

[ref56] Zhou B. Zhong Y. (2022). Instability in the cross-border labor market: a study on the high job turnover of migrant workers from rural Vietnam to rural China. Sustainability 14:e7447. doi: 10.3390/su14127447

